# Supplementary material for: Initial Myeloid Cell Status Is Associated with Clinical Outcomes of Renal Cell Carcinoma
Source: Biomedicines. 2023 Apr 27;11(5):1296. doi: 10.3390/biomedicines11051296 (PMC10216014; doi:10.3390/biomedicines11051296)
Supplement: Supplementary file 1 [file biomedicines-11-01296-s001.zip › biomedicines-2330573-supplementary.pdf]

Table S1. TNM stage and histology of CR, PR, SD, and PD

Patients treated with ICI therapy at first line			n<br>19
Best response (n)		TNM stage	Histology
CR (3)			
	Case 1	T3aN0M1	Bellini duct
	Case 2	T3aN1M1	Acquired cystic disease-associated
	Case 3	T2aN0M1	Clear cell
PR (6)			
	Case 1	T3aN2M1	Clear cell
	Case 2	T3aN0M1	Clear cell
	Case 3	T3aN0M1	Clear cell
	Case 4	T3aN0M1	Clear cell
	Case 5	T3aN0M1	Clear cell
	Case 6	T3aN0M0	Clear cell
SD (5)			
	Case 1	T3aN0M1	Clear cell
	Case 2	T3bN0M1	Clear cell
	Case 3	T3aN0M1	Clear cell
	Case 4	T2N0M1	Clear cell
	Case 5	T3aN2M1	Chromophobe
PD (5)			
	Case 1	T1b N0M1	Clear cell
	Case 2	T1b N0M1	Clear cell
	Case 3	T3cN0M1	Clear cell
	Case 4	T3aN0M1	Clear cell
	Case 5	T3aN0M1	Unclassified

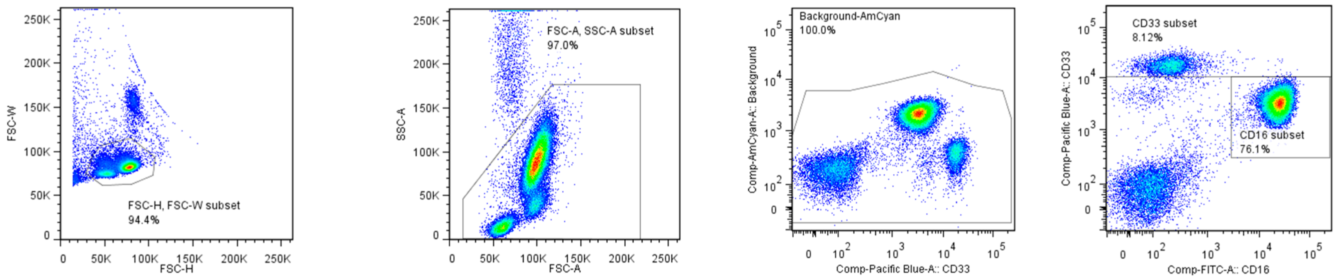

(**a**) (**b**) (c) (d)

Figure S1. Representative gating strategy of flow cytometric analysis of peripheral blood samples. (**a**) The panel of FSC-H vs FSC-W shows the gate for exclusion of aggregated cells. (b) The panel of FSC-A vs SSC-A indicates the gate for exclusion of cell debris. (c) The panel of Pacific Blue-CD33 vs AmCyan-A shows the gate for myeloid cells. (d) The panel of FITC-CD16 vs Pacific Blue-CD33 (D) shows the gate for neutrophilic cells and monocytic cells in peripheral blood.

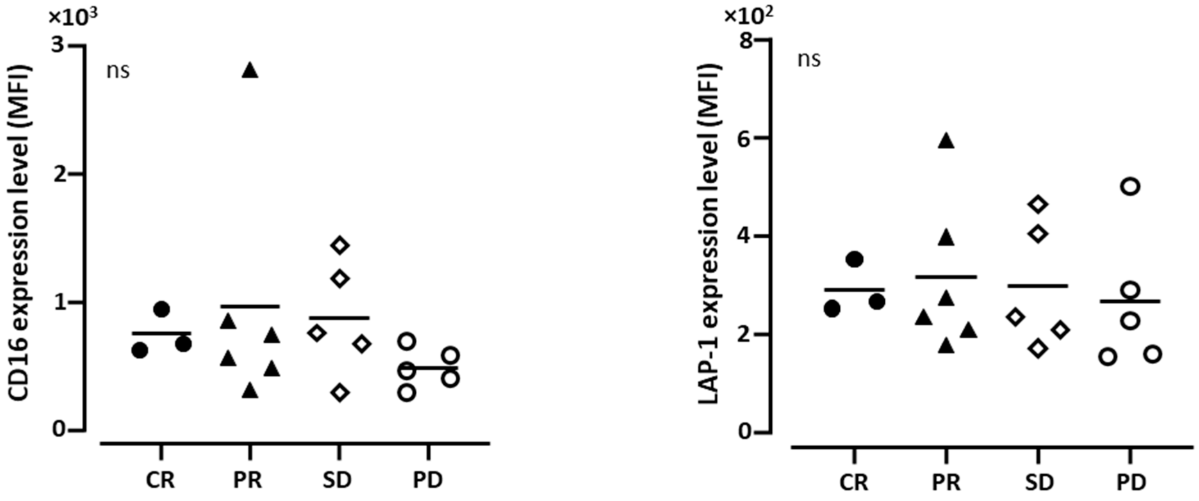

(a) (b)

Figure S2. No characteristic difference in CD16 and LAP-1 expressions immediately before the start of ICI therapy. CD16 (CV; a) and LAP-1 MFI (b) in CD33^{hi} monocytic cells in blood samples were measured by flow cytometry. The blood samples were collected from 1st blood samples (immediately before ICI therapy) patients with RCC, who received ICI therapy as 1st line. The patients were consisting of complete response (CR; n = 3), partial response (PR; n = 6), stable disease (SD; n = 5), and progressive disease (PD; n = 5). Statistical analysis was calculated by non-parametric ANOVA(Kruskal-Wallis) with post hoc test using Dunne, compared to CR vs PR, SD, or PD. Each bar in figure is mean of data; ns, no significant.

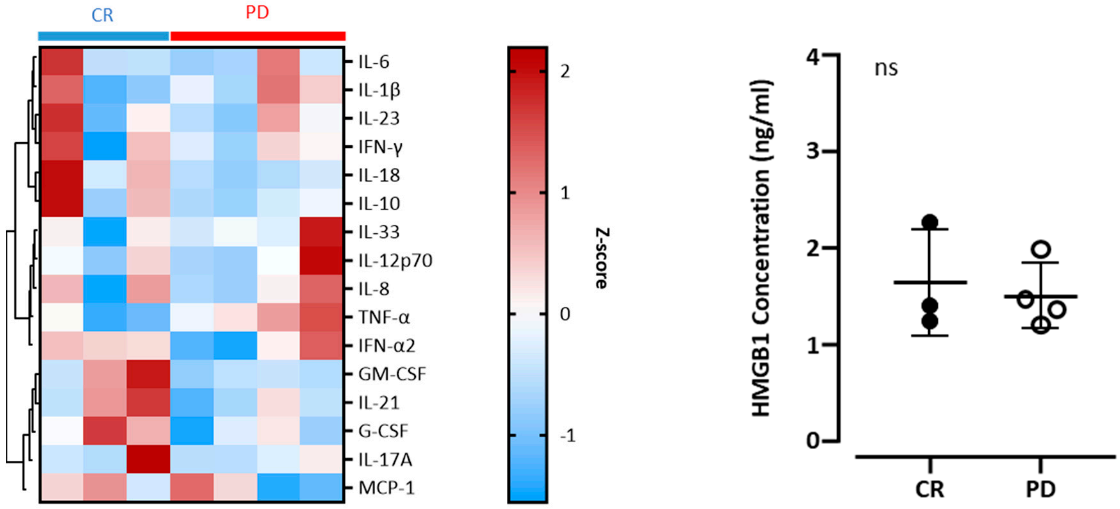

(a) (b)

Figure S3. Absence of relationship between clinical outcomes and pro-inflammatory factors in 2nd blood samples. Plasma collected from 2nd blood sample (after 1st treatment) from patients with complete response (CR) and progressive disease (PD). (a) Heat map of cytokine profile. Cytokines in the plasma were measured by LEGENDplex™ Human Inflammation Panel 1, and then analyzed unsupervised clustering analysis using Z-score distribution with Prism software, (CR, n = 3; PD, n = 4). (b) Comparison of HMGB1 concentrations between patients with CR and PD. HMGB1 in the plasma were measured by ELISA, and data were analyzed using two-tailed unpaired Student's *t*-test. Each data is presented as dot with mean (bar) ± standard deviation (CR, n = 3; PD, n = 4; ns, no significant).
